# Supplementary material for: In Silico Inference of Synthetic Cytotoxic Interactions from Paclitaxel Responses
Source: Int J Mol Sci. 2021 Jan 22;22(3):1097. doi: 10.3390/ijms22031097 (PMC7865701; doi:10.3390/ijms22031097)
Supplement: Supplementary file 1 [file ijms-22-01097-s001.pdf]

Table S1. Lists the count of SC pairs made up by patients. In each group, patients were clustered based on the number of SC pairs, and the order of fewer SC pairs was first.

Rank	Group2 (n)	Group3 (n)	Group4 (n)
1	PRY-TP53 (39)	PRY-TP53 (40)	PRY-TP53 (57)
2	RBMY1J-WWOX (14)	RBMY1J-WWOX (15)	PLEC-ZFY (39)
3	SYNE2-TP53 (5)	CDKN2B-MUC4 (13)	PLEC-RPS4Y1 (39)
4	BPY2-MUC17 (5)	PRORY-MLL2 (12)	PRORY-PLEC (39)
5	ADAM5-CDKN2A (5)	C9orf53-MUC4 (12)	PRORY-MLL2 (39)
6	ADAM3A-CDKN2A (5)	MLL2-RBMY1F (11)	PLEC-SRY (38)
7	DNAH7-HMCN1 (4)	KMT2D-RBMY1F (11)	PLEC-PRKY (38)
8	BPY2B-LCE3C (4)	ADAM5-CDKN2A (11)	PLEC-TXLNG2P (37)
9	SYNE2-TBL1Y (3)	ADAM3A-CDKN2A (11)	EIF1AY-PLEC (37)
10	SON-TP53 (3)	CDKN2B-SYNE1 (9)	CDY1B-PLEC (37)
11	PRORY-SRCAP (3)	CDKN2A-ODZ3 (9)	BCORP1-PLEC (36)
12	PRORY-MLL2 (3)	C9orf53-SYNE1 (9)	PLEC-VCY (34)
13	CDKN2A-RP11 (3)	BCORP1-LAMA1 (9)	MLL2-RBMY1F (33)
14	BCORP1-LAMA1 (3)	BCORP1-COL11A1 (9)	KMT2D-RBMY1F (32)
15	MUC16-WNK1 (2)	SYNE2-TP53 (7)	PLEC-TBL1Y (30)
16	LCE3C-TG (2)	KIAA1797-PRY (7)	AHNAK2-TXLNG2P (30)
17	LCE3B-TG (2)	PRORY-SRCAP (7)	AHNAK2-HSFY2 (30)
18	LCE3B-NRXN1 (2)	COL11A1-TBL1Y (7)	AHNAK2-EIF1AY (30)
19	FLG-UGT2B17 (2)	BPY2B-LCE3C (7)	AHNAK2-PRORY (30)
20	EIF4G1-TBL1Y (2)	SYNE2-TBL1Y (6)	AHNAK2-TXLNGY (30)

Table S2. A multivariate cox proportional hazard analysis of SC burden with pathologic stage.

**BLCA-TP53 Mutant**	**coef**	**exp(coef)**	**se(coef)**	**z**	**Pr(>|z|)**
SC_Burden	−0.924	0.397	0.428	−2.160	0.031
Stage_II	−1.238	0.290	0.377	−3.282	0.001
Stage_III	−0.358	0.699	0.289	−1.238	0.216
Stage_IV	NA	NA	0	NA	NA
**BLCA-TP53 non-Mutant**	**coef**	**exp(coef)**	**se(coef)**	**z**	**Pr(>|z|)**
SC_Burden	0.263	1.30E+00	3.31E-01	0.794	0.427
Stage_II	14.520	2.03E+06	3.95E+03	0.004	0.997
Stage_III	15.560	5.74E+06	3.95E+03	0.004	0.997
Stage_IV	16.330	1.24E+07	3.95E+03	0.004	0.997
**UCEC-TP53 mutant**	**coef**	**exp(coef)**	**se(coef)**	**z**	**Pr(>|z|)**
SC_Burden	−1.440	0.237	0.685	-2.103	0.035
Stage_II	−17.640	0.000	6075	-0.003	0.998
Stage_III	1.036	2.817	0.537	1.929	0.054
Stage_IV	1.669	5.305	0.581	2.871	0.004
**UCEC-TP53 non-mutant**	**coef**	**exp(coef)**	**se(coef)**	**z**	**Pr(>|z|)**
SC_Burden	−0.031	0.970	0.320	−0.096	0.924
Stage_II	1.073	2.925	0.679	1.579	0.114
Stage_III	1.169	3.218	0.552	2.119	0.034
Stage_IV	2.118	8.312	0.696	3.042	0.002
